# Hypoglycaemic activity of Mathurameha, a Thai traditional herbal formula aqueous extract, and its effect on biochemical profiles of streptozotocin-nicotinamide-induced diabetic rats

**DOI:** 10.1186/s12906-017-1851-8

**Published:** 2017-06-29

**Authors:** Kullacha Chayarop, Penchom Peungvicha, Rungravi Temsiririrkkul, Yuvadee Wongkrajang, Wongsatit Chuakul, Piyanuch Rojsanga

**Affiliations:** 1 Department of Pharmaceutical Botany, Faculty of Pharmacy, Mahidol University, 447 Sri-Ayuthaya Road., Rajathevi, Bangkok, 10400 Thailand; 2 Department of Physiology, Faculty of Pharmacy, Mahidol University, 447 Sri-Ayuthaya Road., Rajathevi, Bangkok, 10400 Thailand; 3 Department of Pharmaceutical Chemistry, Mahidol University, 447 Sri-Ayuthaya Road., Rajathevi, Bangkok, 10400 Thailand

**Keywords:** Mathurameha, Thai traditional medicine, Herbal formula, Diabetes mellitus, Hypoglycaemic activity, Acute toxicity

## Abstract

**Background:**

The Thai traditional herbal formula–Mathurameha, consisting of 26 medicinal plants, has been used as an alternative and complementary medicine for diabetes treatment in Wangnamyen Hospital, Thailand. To provide scientific evidences on the efficacy and safety of this herbal formula, in vivo hypoglycaemic activity, effect on serum biochemical profiles and acute toxicity were investigated.

**Methods:**

Experimental type 2 diabetes was induced in male Sprague-Dawley rats by intraperitoneal injection of nicotinamide 15 min prior to intravenous injection of streptozotocin. The most effective extract from the oral glucose tolerant test (OGTT) was administered daily via the oral route to diabetic rats for 2 weeks. Two-hour postprandial plasma glucose (2h–PPG) levels were measured on days 0, 7, and 14. Biochemical data were measured at the end of daily oral administration experiment.

**Results:**

Aqueous extract of the herbal formula was the most potent extract for improving glucose tolerance of streptozotocin-nicotinamide-induced diabetic rats after single oral administration. After 2 weeks of daily oral administration, the aqueous extract showed a dose-dependent glucose lowering effect. At doses of 12.5, 25, and 50 mg/kg, the 2h–PPG level of diabetic rats decreased by 3.32%, 15.78%, and 17.94%, respectively. Most of the biochemical profiles of diabetic rats were improved, including the total cholesterol (TC), alkaline phosphatase (ALP), total protein, albumin, globulin, creatinine, and uric acid levels. The significantly increased triglyceride (TG) level observed in treated diabetic rats indicated a lack of a beneficial effect of the extract on lipid homeostasis. Nevertheless, there were no signs or symptoms of acute toxicity observed after oral administration of aqueous extract (5 g/kg) to both male and female rats.

**Conclusions:**

The results revealed that the herbal formula aqueous extract has hypoglycaemic activity, beneficial effects on biochemical profiles and a lack of acute toxicity. This study confirms the efficacy and safety of the Mathurameha herbal formula used for treating type 2 diabetes mellitus.

## Background

Diabetes mellitus (DM), a chronic metabolic disorder that is characterized by hyperglycaemia, continues to be a major health problem with that has seen a great increase in worldwide incidence due to behaviour and lifestyle changes [1]. In Thailand, there has been a rising trend in the hospitalization rate for patients with diabetes over the last decade, from 380.8 in 2003 to 1081.2 per 100,000 persons in 2013 [2]. Due to high cost of medical care and required treatments with long-term healing, diabetes is considered to be an economic burden and national public health problem that is of great concern [3]. According to the failure of conventional hypoglycaemic drugs to satisfactorily maintain normal glucose levels and some serious side effects [4–6], significant interest in alternative and complementary medicine, especially herbal preparations, has been maintained [7, 8].

Mathurameha, a Thai traditional herbal formula established by Nirund Pongsoiphet and Foundation for the promotion of Thai Traditional Medicine Formula, has long been used by Ayuravedic (Chevagakomarapat) school to treat diabetes mellitus for more than 30 years. In recent years, it has been used as a complementary treatment for type 2 diabetes mellitus in Wangnamyen Community Hospital, Sa kaeo province [9]. The oral dosage form (350 mg capsule) of this herbal formula contains dried powder of 26 medicinal herb combinations **(**Table 1 **)**.

**Table 1:** List of the 26 component herbs of the Mathurameha herbal formula

No.	Scientific name	Family name	Vernacular name	Part used	Authentic specimen collecting location [voucher/collector’s no.]	Commercial crude drug supplier [crude drug’s no.]
1	*Abutilon indicum* (L.) Sweet	Malvaceae	Khrop fan si	Aerial part	Wangnamyen, Sa kaeo, Thailand [PBM 05164]	Herbal drug store [CrDM 0503]
2	*Acanthus ebracteatus* Vahl	Acanthaceae	Ngueak pla mo	Aerial part	Salaya, Nakorn pathom, Thailand [PBM 05186]	Herbal drug store [CrDM 2403]
3	*Andrographis paniculata* (Burm.f.) Nees	Acanthaceae	Fa thalai chon	Aerial part	Rajathevi, Bangkok, Thailand [PBM 05168]	Local herbal farmer [CrDM 1203]
4	*Caryota mitis* Lour.	Arecaceae	Tao rang	Stem	Maerim, Chiang mai, Thailand [QBG 88959]^a^	Herbal drug store [CrDM 0903]
5	*Cyperus rotundus* L.	Cyperaceae	Haeo mu	Rhizome	Rajathevi, Bangkok, Thailand [PBM 04177]	Herbal drug store [CrDM 2503]
6	*Derris reticulata* Craib	Fabaceae	Cha em nuea	Stem	Salaya, Nakorn pathom, Thailand [Chayarop 019]	Herbal drug store [CrDM 0703]
7	*Harrisonia perforata* (Blanco) Merr.	Simaroubaceae	Khon tha	Root	Kabchoeng, Surin, Thailand [PBM 05099]	Herbal drug store [CrDM 0403]
8	*Homalomena aromatica* (Spreng.) Schott	Araceae	Tao kiat	Rhizome	Wangnamyen, Sa kaeo, Thailand [Chayarop 020]	Herbal drug store [CrDM 0803]
9	*Hydnophytum formicarum* Jack	Rubiaceae	Hua roi ru	Rhizome	Maerim, Chiang mai, Thailand [QBG 28200]^a^	Herbal drug store [CrDM 2303]
10	*Imperata cylindrica* (L.) Raeusch.	Poaceae	Ya kha	Root	Maerim, Chiang mai, Thailand [QBG 33404]^a^ Salaya, Nakorn pathom, Thailand [PBM 05188]	Herbal drug store [CrDM 2103]
11	*Lagerstroemia speciosa* (L.) Pers.	Lythraceae	Inthanin nam	Leaf	Rajathevi, Bangkok, Thailand [PBM 05172]	Herbal drug store [CrDM 2603]
12	*Neuropeltis racemosa* Wall.	Convolvulaceae	Sae ma thalai	Stem	Wangnamyen, Sa kaeo, Thailand [PBM 05111]	Local herbalist [CrDM 2003]
13	*Orthosiphon aristatus* (Blume) Miq.	Lamiaceae	Ya nuat maeo	Aerial part	Rajathevi, Bangkok, Thailand [PBM 05171]	Herbal drug store [CrDM 2203]
14	*Pandanus odorifer* (Forssk.) Kuntze	Pandanaceae	Lam chiak	Root	Maerim, Chiang mai, Thailand [QBG 24201]^a^	Herbal drug store [CrDM 1603]
15	*Parameria laevigata* (Juss.) Moldenke	Apocynaceae	Muak khao	Stem	Loengnokta, Yasothon, Thailand [PBM 02752]	Herbal drug store [CrDM 1303]
16	*Premna herbacea* Roxb.	Lamiaceae	Khao yen tai	Rhizome	Li, Lamphun, Thailand [QBG 3907]^b^	Herbal drug store [CrDM 0203]
17	*Rhinacanthus nasutus* (L.) Kurz	Acanthaceae	Thong phan chang	Aerial part	Rajathevi, Bangkok, Thailand [PBM 05170]	Herbal drug store [CrDM 1003]
18	*Salacia chinensis* L.	Celastraceae	Kam phaeng chet chan	Stem	Kantararom, Srisaket, Thailand [PBM 04927]	Herbal drug store [CrDM 0103]
19	*Smilax glabra* Roxb.	Smilacaceae	Khao yen nuea	Rhizome	Wang chin, Phrae, Thailand [QBG 3138] ^b^	Herbal drug store [CrDM 0303]
20	*Solanum trilobatum* L.	Solanaceae	Ma waeng khruea	Fruit	Wangnamyen, Sa kaeo, Thailand [PBM 05166]	Herbal drug store [CrDM 1503]
21	*Terminalia bellirica* (Gaertn.) Roxb.	Combretaceae	Samo phi phek	Ripen fruit	Kabchoeng, Surin, Thailand [PBM 04172]	Herbal drug store [CrDM 1903]
22	*Terminalia chebula* Retz.	Combretaceae	Samo thai	Ripen fruit	Kabchoeng, Surin, Thailand [PBM 04173]	Herbal drug store [CrDM 1803]
23	*Terminalia* sp.	Combretaceae	Samo thet	Ripen fruit	N/A	Herbal drug store [CrDM 1703]
24	*Tinospora crispa* (L.) Hook. f. & Thomson	Menispermaceae	Bora phet	Stem	Wangnamyen, Sa kaeo, Thailand [PBM 04215]	Herbal drug store [CrDM 1103]
25	*Tribulus cistoides* L.	Zygophyllaceae	Khok kra sun	Aerial part	Baan lad, Phetburi, Thailand [PBM 05163]	Herbal drug store [CrDM 0603]
26	*Urceola minutiflora* (Pierre) Mabb.	Apocynaceae	Muak daeng	Stem	Maerim, Chiang mai, Thailand [QBG 41448] ^a^	Herbal drug store [CrDM 1403]

^a^Specimens collected from Queen Sirikit Botanic Garden (QSBG) under the regulation for accessing biological resources; ^b^ Specimens received from Assoc. Prof. Weena Jiratchariyakul

From previous studies, in vivo hypoglycaemic effects of 12 component herbs have been reported [10–32], and 8 component herbs have been studied for their hypoglycaemic actions, including α-glucosidase inhibitory activity [30, 31, 33–38], inhibition of glucose absorption [17], stimulation of insulin secretion from pancreatic β-cells [17, 24, 29, 39], stimulation of glucose uptake to fat and muscle cells [18, 40–44], normalization of hepatic glucose output and glycogen synthesis [19, 43]. Twelve active hypoglycaemic compounds have been identified — these include andrographolide from *Andrographis paniculata* (Burm.f.) Nees [30, 45]; corosolic acid [36] and lagerstroemin [41, 42] from *Lagerstroemia speciosa* (L.) Pers; salacinol and kotalanol from *Salacia chinensis* (L.) [31]; gallic acid from *Terminalia bellirica* (Gaertn.) Roxb. [21]; chebulanin, chebulagic acid and chebulinic acid from *Terminalia chebula* Retz. [34]; and borapetoside C [35, 43] and borapetol B [39] from *Tinospora crispa* (L.) Hook.f. & Thomson. Therefore, the hypoglycaemic effects as well as the mechanism of actions of this anti-diabetic herbal formula are worth investigating.

Because of the high oral solid dosage form of this formula (5 capsules twice daily) as well as the unappreciable taste of the decoction, development of a preparation based on traditional knowledge might be helpful to reduce the administration dose [46]. Therefore, a comparison between the hypoglycaemic effects of the dried powder and extracts of the whole formula is needed to confirm the efficacy of the extract and to examine the most suitable extraction method. Although this herbal formula has already been used in diabetic patients without any sign or symptom of toxicity [9], the toxicity study of the whole formula extract remains necessary to ensure its safety for further usage.

The aims of this study are to observe the in vivo hypoglycaemic activity, effects on biochemical profiles as well as acute toxicity of the whole formula extracts to provide scientific evidence of its efficacy and safety, which might be useful for the further development of this herbal drug as well as for treating diabetes patients with fewer undesirable side effects.

## Methods

### Chemicals

Nicotinamide, o-dianisidine, PGO enzyme and streptozotocin (STZ) were purchased from Sigma Chemical Co. (St Louis, MO, USA); Glibenclamide tablets (Daonil) were purchased from Aventis Pharma Ltd. (Bangkok, Thailand). All other chemicals and reagents were of analytical grade.

### Plant materials

Commercial crude drug specimens of 26 single herbs were sampled from dried plant materials, which were used in Wangnamyen Hospital from February 2012 to November 2013. All of the drug specimens were deposited at the Department of Pharmaceutical Botany, Faculty of Pharmacy, Mahidol University (Crude drug’s no. are shown in Table 1). The commercial crude drug specimens of each herb were verified their scientific names by comparing the microscopic characteristics and thin-layer chromatographic fingerprints to the authentic crude drug specimens.

Authentic crude drug specimens of 17 herbs were collected from different locations in Thailand. All of the authentic specimens were identified according to the standard botanical literature and compared with specimens of the herbarium collection at the Forest Herbarium (BKF), Department of National Parks, Wildlife and Plant Conservation. Then, they were deposited at Mahidol University Herbarium (PBM), Department of Pharmaceutical Botany, Faculty of Pharmacy, Mahidol University. The authentic specimens of 7 herbs were collected from Queen Sirikit Botanic Garden (QSBG) under the regulations for accessing biological resources, while the other 2 authenticated crude drugs were gifts from from Assoc. Prof. Weena Jiratchariyakul. The voucher numbers of all of the authentic specimens are shown in Table 1.

*Derris reticulata* and *Homalomena aromatica* reproductive parts were not collected. As a result, they were identified by comparing the characteristics of their vegetative parts with the standard botanical literature as well as an organoleptic examination compared with a Thai traditional medicine textbook [47]. The crude drug, Samo thet, was considered to be a member of *Terminalia* genus according to its fruit characteristics [48].

### Extractions

Crude drugs of 26 single herbs were mixed and ground together. The whole formula powder was then separately extracted by 3 different solvents — 80% ethanol, 50% ethanol, or water. Ethanolic extracts were achieved by percolation; the miscella was dried by a rotary evaporator. The percentage yield of 80% ethanol and 50% ethanol extracts were 24.57% and 28.12% dry weight, respectively. Aqueous extract was derived from a decoction of the powdered mixture (1 kg) by soaking with water for 30 min and then boiling at 100 °C for 30 min. The decoction was filtered through gauze and dried by a spray dryer, yielding 14.73% dry weight. All extracts were kept in closed containers at 4 °C until use.

### Animal maintenance

Sprague-Dawley rats (105 male and 5 female rats), weighting 120–150 g, were purchased from the National Laboratory Animal Centre (NLAC), Mahidol University, Thailand. They were housed in an air conditioned room (22–25 °C) and subjected to a 12-h light/dark cycle for at least 1 week prior to the experiment. The animals were given free access to a pellet diet and water ad libitum. The animal protocol was approved by the Animal Care and Used Committee, Faculty of Pharmacy, Mahidol University, Thailand (PYT008/2555).

### Induction of experimental diabetes

Experimental type 2 diabetes was induced in rats using the method in Masiello et al. [49], with a slight modification. Overnight fasted male rats were intraperitoneally injected with 80 mg/kg nicotinamide 15 min before a single intravenous injection of 65 mg/kg streptozotocin (STZ). The urine glucose levels were checked weekly with a urine glucose strip. Three weeks after induction, the 6-h fasting plasma glucose (FPG) levels were examined, and the rats that had a FPG level greater than 160 mg/dL were used in further experiments.

### In vivo hypoglycaemic experiments

#### Effect on oral glucose tolerance of normal and diabetic rats

The dosages of all of the extracts (mg/kg by weight) used in this study were calculated from the percent yield of extractions equivalent to 350 mg of the dry powdered mixture. All of the extracts and glibenclamide were dissolved in distilled water. The ethanolic extracts were then sonicated for 15 min. The dry powdered mixture was suspended in distilled water with vigorous stirring and was immediately given to the rats.

Overnight fasted normal male rats were randomly divided into 6 experimental groups with 5 rats per group. The oral glucose tolerant test (OGTT) was performed according to Peungvicha et al. [50]. Blood samples of all rats were collected by drawing from the tail vein, and each group was immediately given oral treatments (assigned as −30 min) as follows:

Group 1: distilled water (negative control).

Group 2: glibenclamide, 5 mg/kg.

Group 3: 80% ethanol extract, 87.5 mg/kg.

Group 4: 50% ethanol extract, 98 mg/kg.

Group 5: aqueous extract, 50 mg/kg.

Group 6: powdered mixture suspension, 350 mg/kg.

Thirty minutes later (assigned as 0 min), blood samples were collected and glucose (1.25 g/kg b.w.) was immediately given to all rats by oral administration. After glucose loading, blood samples were collected every 30 min until 150 min. The plasma glucose level of each time point was examined by the glucose oxidase method.

Diabetic rats were fasted 6 h prior to the experiment and were randomly divided into 6 experimental groups (5 rats per group), which were then treated with the same treatment as normal rats. The OGTT experiment was performed with the same protocol as that explained above. The most effective extract was selected for further experiments.

#### Effects on the postprandial plasma glucose levels

STZ-nicotinamide-induced diabetic rats were randomly divided into 8 experimental groups, with 5 rats per group. Each group was daily orally given with distilled water, glibenclamide, various doses of aqueous extract or powdered mixture suspension for 2 weeks as follows:

Group 1: distilled water (diabetic control).

Group 2: glibenclamide, 5 mg/kg b.w.

Group 3–5: aqueous extract, 12.5, 25, and 50 mg/kg.

Group 6–8: powdered mixture suspension, 87.5, 175, and 350 mg/kg.

Two-hour postprandial plasma glucose (2h–PPG) levels were determined on days 0 (the day before oral administration started), 7, and 14. Blood samples were collected from the tail vein after 2 h of fasting and were examined for the plasma glucose level by the glucose oxidase method. The rats were weighted on days 0, 7 and 14 to adjust the treatment doses [18].

### Effect on the serum biochemical profiles of diabetic rats

At the end of the 2-week daily oral administration and postprandial plasma glucose experiment, each rat was euthanized prior to blood sample drawing from the heart. The blood samples were centrifuged at 5000 rpm for 10 min; then, blood serum was separated. All serum samples were kept at −20 °C until further examinations. Diabetic rats that were orally given distilled water served as the ‘diabetic control’ group, while normal rats that received oral administration of distilled water served as the ‘normal control’ group.

The biochemical profiles, including alanine transferase (ALT), aspartate transferase (AST), alkaline phosphatase (ALP), total protein, albumin, globulin, blood urea nitrogen (BUN), creatinine, uric acid, total cholesterol (TC) and triglycerides (TG), were measured by National Laboratory Animal Centre (NLAC), Mahidol University, Thailand.

### Acute toxicity

The aqueous extract and powdered mixture suspension that dissolved in distilled water was orally administered to male and female rats (*n* = 5 in each sex) at a dose of 5 g/kg. The animals were observed at least once during the first 30 min and periodically during the first 24 h following a standard behavioural, neurologic, and autonomic animal profile. Afterwards, they were observed daily for a total of 14 days for any indication of illness or death. Food and water were provided ad libitum.

### Statistical analysis

The distribution of data was analysed by Komogorov-Smirnof’s normality test. The datasets with a normal distribution were then analysed by one-way ANOVA and/or Student’s t-test. The datasets with an abnormal distribution were analysed by the Mann-Whitney test. A *p*-value less than 0.01 and/or 0.05 indicated a significantly difference.

## Results

### Effects on the oral glucose tolerance of normal and diabetic rats

**Fig. 1:**
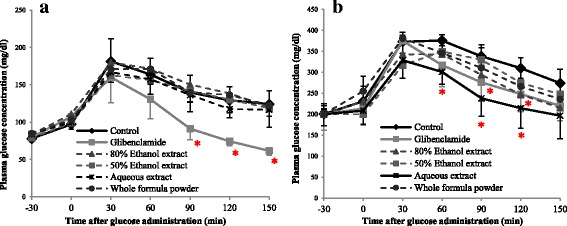
Effects of the whole formula powder and extracts on oral glucose tolerance of **a** normal rats and **b** diabetic rats (*N* = 5); *Significant different from control group (*p < 0.05*; Student’s t-test); Data were expressed as mean ± S.E

The effects of the whole formula powder and extracts on the oral glucose tolerance of normal rats are shown in Fig. 1a. The plasma glucose level of all of the extract treated groups was not different from that of the control group while the glibenclamide-treated group showed a significant decrease in plasma glucose levels at 90–150 min after glucose administration. In diabetic rats, whole formula powder and extracts improved their glucose tolerance, as shown in Fig. 1b.

Both ethanolic extracts (80% and 50% ethanol extracts) and powdered mixture suspensions showed a similar potency in lowering the plasma glucose levels. Aqueous extract significantly lowered the plasma glucose levels of treated rats at 60–120 min after glucose administration, as well as after glibenclamide administration, compared to the control group. Therefore, aqueous extract was selected for further experiments [51].

### Effects on the postprandial plasma glucose levels

After 1-week of daily oral administration, diabetic rats treated with 50 mg/kg aqueous extract showed a significant decrease in the 2-hour postprandial plasma glucose (2h–PPG) level of 19.96%, while the other doses had no effects on the 2 h–PPG levels. After 2 weeks, aqueous extract at doses of 12.5, 25 and 50 mg/kg decreased the 2h–PPG levels of diabetic rats in a dose-dependent manner by 3.32%, 15.78% and 17.94%, respectively [51]. However, the effects of equivalent doses of the powdered mixture suspension did not correspond to aqueous extract due to the lack of a dose-dependent pattern.

**Fig. 2:**
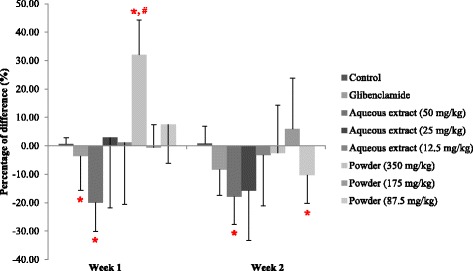
Effect of herbal formula aqueous extract and powder on 2-h postprandial plasma glucose (2h–PPG) levels of STZ-induced diabetic rats (*N* = 5); *Significant different from control group of each week; #Significant different from glibenclamide group of each week (*p < 0.05*; Mann-Whitney); Data were expressed as mean ± S.E.

After a significant increase in the 2h–PPG level by 31.97% in the first week, 350 mg/kg powdered mixture suspension slightly decreased the 2h–PPG levels of rats after 2-weeks of administration. At a dose of 175 mg/kg, the 2h–PPG levels of diabetic rats were not changed in the first week; instead, they were slightly increased in the second week. The two-hour PPG levels of diabetic rats treated with 87.5 mg/kg powdered mixture suspension were slightly increased in the first week, but they were significant decreased by 10.26% in the second week (Fig. 2).

### Effects on the serum biochemical profile of diabetic rats

The results from serum biochemical profiles determinations are shown in Table 2. Data were expressed as the mean ± S.D. of each group.

**Table 2:** Serum biochemical profiles of streptozotocin-nicotinamide-induced diabetic rats treated with various doses of the aqueous extract and whole formula powder of Mathurameha herbal formula

Treatment group (*N* = 5)	Lipid profiles		Liver functions						Renal functions		
	CHOL (mg/dL)	TG (mg/dL)	ALP(U/L)	ALT(U/L)	AST(U/L)	TP(g/dL)	ALB(g/dL)	GLO(g/dL)	BUN(mg/dL)	CREA(mg/dL)	UA (mg/dL)
Normal control	96.80 ± 4.55^b^	93.40 ± 14.66	85.40 ± 14.71^b^	57.80 ± 9.73	122.40 ± 34.05	7.42 ± 0.24^b^	4.90 ± 0.10^b^	2.54 ± 0.18^b^	19.78 ± 1.80^b^	0.28 ± 0.04^b^	2.2 ± 0.56^b^
Diabetic control	119.20 ± 15.32^a^	138.00 ± 39.34	541.80 ± 167.86^a^	61.60 ± 19.59	106.60 ± 17.95	5.38 ± 0.33^a^	3.58 ± 0.30^a^	1.80 ± 0.14^a^	33.18 ± 5.88^a^	0.36 ± 0.05^a^	4.54 ± 2.15^a^
Glibenclamide 5 mg/kg	97.20 ± 23.51^b^	132.80 ± 32.52	388.00 ± 144.73^a^	52.00 ± 34.12	96.60 ± 50.28	5.00 ± 0.79^a^	3.56 ± 0.50^a^	1.42 ± 0.34^a^	30.76 ± 6.13^a^	0.28 ± 0.04^b^	2.48 ± 1.93^b^
Aqueous extract 12.5 mg/kg	125.25 ± 13.77^a^	197.00 ± 102.36^a^	289.00 ± 94.26^b^	62.00 ± 21.35	117.25 ± 34.51	6.98 ± 0.90^b^	4.78 ± 0.43^b^	2.25 ± 0.62	34.08 ± 8.04^a^	0.30 ± 0.08^b^	2.50 ± 0.93^b^
Aqueous extract 25 mg/kg	100.40 ± 5.98^b^	154.40 ± 43.17	315.40 ± 132.89^ab^	30.80 ± 14.91^ab^	84.40 ± 29.99	5.32 ± 0.59^a^	3.54 ± 0.23^a^	1.74 ± 0.38^a^	30.86 ± 5.42^a^	0.26 ± 0.05^b^	1.46 ± 0.34^b^
Aqueous extract 50 mg/kg	110.80 ± 21.66	168.00 ± 66.12^a^	384.40 ± 173.95^a^	43.80 ± 12.93	98.60 ± 30.78	6.04 ± 1.16^a^	4.02 ± 0.79^a^	1.98 ± 0.52^a^	33.82 ± 10.60^a^	0.26 ± 0.05^b^	2.34 ± 1.35^b^
Whole formula powder 87.5 mg/kg	112.50 ± 14.53^a^	181.25 ± 31.53^a^	413.00 ± 197.22^a^	41.00 ± 13.44	94.50 ± 49.68	6.10 ± 0.42^a^	4.20 ± 0.18^ab^	1.90 ± 0.34^a^	39.00 ± 13.26^a^	0.23 ± 0.05^b^	1.80 ± 0.80^b^
Whole formula powder 175 mg/kg	121.75 ± 20.85^a^	168.25 ± 67.37^a^	398.75 ± 208.32^a^	47.50 ± 11.73	109.25 ± 33.09	6.88 ± 1.24^b^	4.68 ± 0.51^b^	2.25 ± 0.69	37.38 ± 10.15^a^	0.33 ± 0.05^b^	2.55 ± 2.32^b^
Whole formula powder 350 mg/kg	119.00 ± 12.10	141.80 ± 33.49	465.80 ± 222.82^a^	48.60 ± 14.94	110.40 ± 25.86	5.92 ± 0.46^a^	4.16 ± 0.22^a#^	1.80 ± 0.31^a^	35.10 ± 4.25^a^	0.24 ± 0.05^b^	2.78 ± 1.20^b^

Data were presented as a mean ± S.D.; *CHOL* total cholesterol, *TG* triglyceride, *ALP* alkaline phosphatase, *ALT* alanine transferase, *AST* aspartate transferase, *TP* total protein, *ALB* albumin, *GLO* globulin, *BUN* blood urea nitrogen, *CREA* creatinine, *UA* uric acid;

^a^Significant different from normal control, ^a^Significant different from diabetic control (*p < 0.05*; One-way ANOVA)

#### Effects on lipid profiles

Aqueous extract at a dose of 25 mg/kg significantly decreased the total cholesterol (TC) level of diabetic rats (15.8%), while 50 mg/kg of the extract slightly decreased the TC level (7.05%) compared to the diabetic control. The whole formula powder had no effect on the TC level of diabetic rats. Both aqueous extract and the whole formula powder increased the triglycerides levels of diabetic rats compared to normal controls.

#### Effects on the liver function

Aqueous extract at doses of 12.5 and 25 mg/kg significantly lowered the serum level of alkaline phosphatase (ALP) (46.7% and 41.8%, respectively), while 50 mg/kg slightly, but not significantly, lowered the level (29.0%) of diabetic rats compared with the diabetic control group. The serum levels of alanine transferase (ALT) and aspartate transferase (AST) in the diabetic control group were not significantly different from those of the normal control group. However, aqueous extract at a dose of 25 mg/kg significantly decreased the ALT level and slightly decreased the AST levels of diabetic rats. Aqueous extract at a dose of 12.5 mg/kg and the powdered mixture suspension at a dose of 175 mg/kg significantly increased the total protein and albumin and slightly increased the globulin levels of diabetic rats compared to diabetic controls.

#### Effects on the renal function

The serum levels of blood urea nitrogen (BUN), creatinine, and uric acid of diabetic rats were significantly higher than those of the normal control group. The herbal formula had no effect on the BUN level of treated diabetic rats. By contrast, the serum creatinine and uric acid levels of diabetic rats treated with both aqueous extract and powdered mixture suspension significantly decreased to normal levels.

### Acute toxicity

Aqueous extract and powdered mixture suspension, at a dose of 5 g/kg, when given to male and female rats, showed no effect on their behavioural, neurologic, and autonomic profiles during the 24 h observation periods. No mortality was observed for up to 14 days of monitoring. The value of the lethal dose at 50% of the extract in rats was estimated to be more than 5 g/kg.

## Discussion

In this study, diabetes in rats was induced by a streptozotocin-nicotinamide injection. Streptozotocin (STZ) has toxic effects on pancreatic β-cells based on its potent alkylating properties, which are related to cell death induction. STZ generates reactive oxygen species and acts as a nitric oxide donor, which contributes to DNA fragmentation and caused other changes that resulted in the destruction of pancreatic islet cells [52, 53]. The administration of suitable dosages of nicotinamide partially protects β-cells from the cytotoxic effect of STZ. Therefore, the diabetes syndrome induced in this experiment appears to be closer to human type 2 diabetes in that it demonstrated a significant response to glucose and sensitivity to sulfonylureas, a class of hypoglycaemic drug [49]. These drugs, including glibenclamide, stimulate insulin secretion from pancreatic β-cells by closing the ATP-dependent potassium channel (K_ATP_) [54].

The oral glucose tolerance test (OGTT), which examines changes in the plasma glucose levels in response to oral glucose administration, has long been used clinically for diabetes mellitus diagnosis and in research to evaluate the effectiveness of hypoglycaemic agents [17]. In the present study, the OGTT was employed in a single dose administration experiment to determine the plasma glucose lowering effects of the Mathurameha herbal formula and its extracts at the same equivalent dose of 350 mg/kg in both normal and diabetic rats.

In normal rats, the plasma glucose levels of all of the treated groups and the untreated control group at every time point after glucose administration were the same. This indicates that the herbal formula did not alter blood glucose regulation in normoglycaemic conditions. However, the hypoglycaemic effects of the herbal formula were observed in diabetic rats. A single dose of aqueous extract (50 mg/kg) exhibited outstanding plasma glucose lowering effects in diabetic rats **(**Fig. 1 **)**. The results suggested that the major components that possessed hypoglycaemic activity of this herbal formula could be polar compounds, which are soluble in water. Daily oral administration of various doses of aqueous extract to diabetic rats revealed that a dose of 50 mg/kg exhibited the most potent glucose lowering effect and lowered 2-hour postprandial plasma glucose (2h–PPG) level after a 1-week administration, and this effect remained after 2-week administration. While the powdered mixture suspension showed a non-dose dependent glucose lowering effect, aqueous extract exhibited this effect in a dose-dependent manner after 2 weeks **(**Fig. 2 **)**, possibly because of the difference in the intestinal absorption of actively hypoglycaemic components. In the powdered mixture suspension, the active components that are covered by plant cell wall lead to low consistency in intestinal absorption. By contrast, the active components in aqueous extract are more available for absorption, resulting in high consistency in intestinal absorption. Aqueous extract is preferred for developing an herbal drug formulation for diabetes treatment.

Furthermore, previous reports showed that aqueous extract of medicinal plants, including *Abutilon indicum* [17, 28], *Derris reticulata* [20], *Lagerstroemia speciosa* [16, 55], *Orthosiphon aristatus* [29], *Solanum trilobatum* [14], *Terminalia chebula* [23], and *Tinospora crispa* [24], which are the components of this herbal formula, have in vivo hypoglycaemic effects. It has been reported that *Lagerstroemia speciosa* contains polar active compounds, including ellagitannins, such as lagerstroemin and ellagic acid derivatives, which could stimulate glucose uptake into 3T3-L1 adipocytes [40–42]. Ellagitanins from *Terminalia chebula* including chebulanin, chebulagic acid, and chebulinic acid, have been reported to have inhibitory effects against α-glucosidase [34]. Boropetoside C, which was isolated from *Tinospora crispa*, also showed α-glucosidase inhibitory effects [35], increased glucose utilization in peripheral tissues, and reduced hepatic gluconeogenesis [43]. Therefore, it is suggested that these compounds might contribute to the hypoglycaemic effects of this herbal formula aqueous extract.

In this study, an increase in the serum level of total cholesterol (TC) triglycerides (TG) was observed in diabetic rats. The elevation of the TC and TG levels in circulation could be due to the increase in cholesterogenesis, fatty acid uptake, and deposit as triglycerides in the liver [56, 57]. The altered TC levels were significantly restored to normal values by aqueous extract (25 mg/kg) treatment **(**Table 2 **)**. The extract might have hypocholesterolomic effects with clearance elevation and reduction of endogenous cholesterol production [57, 58]. However, an increase in the serum TG level was observed in diabetic rats that were treated with extract **(**Table 2 **)**. This indicates that the formula failed to reduce TG production and could not control mobilization of triglycerides from the liver to circulation [59].

Liver enzymes, including AST, ALT, and ALP, in circulation that were investigated in this study are indicators of hepatic damage as they are released into the bloodstream after cell membrane damage reflecting hepatocellular necrosis [57, 60, 61]. However, the serum AST and ALT levels of the diabetic rats used in this study were not significantly different from those of the normal control group **(**Table 2 **)**. This might be due to the protection of nicotinamide against the hepatotoxic effects of STZ [62, 63]. Moreover, the significant decrease in the ALT levels in the 25 mg/kg aqueous extract treated group might be from the gluconeogenesis inhibitory effects of the extract [64]. Nevertheless, the serum ALP levels of the diabetic control group were significantly increased, indicating leakage from liver damage. A decline in the serum ALP levels was observed in the diabetic groups that were treated with aqueous extract (12.5 and 25 mg/kg) **(**Table 2 **)**. This revealed that the extract could reduce the leakage of liver enzymes into the bloodstream.

The decline of plasma proteins in diabetes, albumin and globulin, which are mainly synthesized by the liver, might be from the augmentation of protein catabolism or microproteinuria, indicators of diabetic nephropathy [64]. This study demonstrated that the serum total protein, albumin, and globulin levels of diabetic rats were significantly lower than those of the normal control group. The significant increase in these serum proteins was observed in diabetic groups that were treated with the 12.5 mg/kg aqueous extract **(**Table 2 **)**. These results are in agreement with decreasing the serum ALP levels by aqueous extract, indicating the protective effects of herbal formulas against liver damage that are associated with hyperglycaemia. Considering the evidence implicating diabetes mellitus in hepatic dysfunction, this hepatoprotective effect may be due to the hypoglycaemic effects of the whole formula aqueous extract investigated in this study [65].

Diabetic hyperglycaemia induces elevation of serum blood urea nitrogen (BUN), creatinine, and uric acid, which are considered to be significant markers of renal dysfunction [66, 67]. A significant increase in the serum BUN, creatinine and uric acid levels was observed in diabetic rats compared to the normal control group. Aqueous extract could not restore the altered BUN levels in diabetic rats **(**Table 2 **)**. This might indicate that the extract could not reduce the accumulation of urea nitrogen that occurred due to the enhanced breakdown of both liver and plasma proteins and that it did not play a role in protein metabolism [68]. Additionally, the treatment with all doses of aqueous extract significantly reduced the serum creatinine and uric acid levels of diabetic rats to normal levels **(**Table 2 **)**. This indicates improvement of the kidney excretion ability of creatinine, a byproduct of the breakdown of creatine and phosphocreatine, which are energy storage compounds in muscle, as well as uric acid, an end product of purine metabolism [68]. The normalization of the serum creatinine and uric acid levels indicates the role of this herbal formula in renal function improvement [67].

Overall, the results suggested that the aqueous extract is a good candidate for diabetes treatment because no undesirable effects on liver and kidney functions were observed, as it could be generated by long-term treatment with anti-diabetic agents. Although, the most potent dose of aqueous extract in regard to hypoglycaemic activity was 50 mg/kg, lower doses (12.5 and 25 mg/kg) exhibited more beneficial effects on the lipid profiles and liver and kidney functions. Therefore, 25 mg/kg was considered to be the most suitable dose because it has both potent hypoglycaemic activity and biochemical profile improvement effects in diabetic rats. Therefore, this dose should be used in the experimental design of further pharmaceutical, pharmacological and clinical studies on this herbal formula.

Aqueous extract did not exhibit acute toxicity to either sex of normal rats. Its LD_50_ was estimated to be higher than 5 g/kg. This result agreed with those of previous studies that reported the aqueous extracts of *Abutilon indicum* [17, 69], *Acanthus ebracteatus* [70], *Harrisonia perforata* [71], *Imperata cylindrica* [13], and *Terminalia chebula* [72] showed no acute toxicity in animals.

## Conclusion

The plasma glucose lowering effect in both single and daily repeated oral administration of whole formula aqueous extract observed in STZ-nicotinamide-induced diabetic rats revealed its potent hypoglycaemic activity. Overall, the results suggested that aqueous extract is a good candidate for diabetes treatment due to the lack of observed undesirable effects on liver and kidney functions. The extract was not only unaltered, it improved most biochemical profiles, including total cholesterol, alkaline phosphatase, total proteins, albumin, globulin, creatinine, and uric acid. It was considered to have hypocholesterolomic, hepatoprotective, and renoprotective effects during diabetic hyperglycaemia. However, the elevation of serum triglycerides levels of diabetic rats treated with aqueous extract should be considered. Aqueous extract has no acute toxicity on male and female rats at a dose of 5 g/kg.

This study confirms the efficacy and safety of the Mathurameha herbal formula treating type 2 diabetes mellitus. The revealed hypoglycaemic activity, beneficial effects to biochemical profiles and non-acute toxicity of the entire formula aqueous extract provided valuable insight for next-steps research of this herbal formula. Further investigations on the in vitro hypoglycaemic actions of the whole formula aqueous extract and chemical compounds responsible for its effect should be performed to clarify the mechanisms and active compounds.

## Abbreviations

2h–PPG: 2-h postprandial plasma glucose
ALP: Alkaline phosphatase
ALT: Alanine transferase
AST: Aspartate transferase
BUN: Blood urea nitrogen
FPG: Fasting plasma glucose
OGTT: Oral glucose tolerant test
PGO: Peroxidase-glucose oxidase
STZ: S treptozotocin
TC: T otal cholesterol
TG: Triglyceride
